# Innovative Development of Research Engagement Manual: Strategies to Enhance Recruitment and Retention of Black Individuals in Clinical Trials for Substance Use Disorders

**DOI:** 10.1177/24731242251362181

**Published:** 2025-08-22

**Authors:** Geoffrey Obel, DeWanda Harris-Trimiar, Joshua S. Elmore, Taryn L. Mayes, Adrienne Mays, Angela Casey-Willingham, Srividya Vasu, Steven Shoptaw, Madhukar H. Trivedi

**Affiliations:** ^1^ Department of Preventative Medicine and Population Health, UT Tyler Health Science Center, Tyler, Texas, USA.; ^2^ North Central Texas Council of Governments, Arlington, Texas, USA.; ^3^ Center for Depression Research and Clinical Care, Peter O’Donnell Jr. Brain Institute and Department of Psychiatry, University of Texas Southwestern Medical Center, Dallas, Texas, USA.; ^4^ Department of Psychiatry and Biobehavioral Sciences, University of California, Los Angeles, California, USA.

**Keywords:** Black individuals, health equality, diversity, substance abuse, participation barriers

## Abstract

**Background:**

The participation of Black individuals in clinical trials remains lower than that of other racial and ethnic groups. Substance abuse adds additional barriers to recruitment and retention. While significant attention has been devoted to identifying barriers to recruitment/retention, efforts have been largely unsuccessful in increasing the participation of Black individuals in clinical trials. This article details Phase 1 efforts to develop sustainable strategies to increase enrollment/recruitment of Black participants in clinical trials through an Innovative Development of Research Engagement Manual.

**Methods:**

Phase 1 involved a literature review and the establishment of an Expert Diversity Advisory Board, which identified barriers to Black individual participation in substance use disorder (SUD) research. Identified barriers included lack of awareness of research, mistrust, lack of comfort with research, lack of information, and time/resource constraints. Focus groups were conducted to assess the importance of the identified factors in 61 Black participants with SUD history.

**Results:**

Among the focus group participants, 37.7% indicated mistrust, 45.9% indicated a lack of knowledge, and 27.9% stated safety concerns as reasons for not engaging with researchers. They considered compensation, research benefits, study duration, privacy, safety, and side effects as vital information that informed their decisions on clinical trial participation. The focus groups identified financial incentives, potential treatment options, and potential for improved awareness about substance abuse treatment as factors that determine retention in a study.

**Conclusion:**

With barriers identified, future efforts will focus on qualitative assessments of focus group material and developing and evaluating the manual.

## Introduction

Racial and ethnic disparities in health care, clinical research participation, and health outcomes remain persistent in the United States. Racial and ethnic minority groups exhibit a disproportionately high burden of chronic diseases but are underrepresented in clinical research. In 2014, the Food and Drug Administration introduced a 5-year action plan to improve the participation of diverse populations in clinical trials, with mandatory annual reporting of drug trials snapshot summaries.^1^ Despite efforts to monitor and encourage the representation of ethnic minorities in clinical trials, the 2023 Drug Snapshot Summary report shows that their participation is still low compared with White individuals, especially for Black individuals, with representation over 10% in only nine drug programs.^2^ Underrepresentation of ethnic minorities in clinical trials has significant clinical implications and ultimately compromises the generalizability of research findings, further exacerbating existing disparities. The challenges of recruiting Black individuals for clinical trials have been discussed previously in medical conditions such as cancer, cardiovascular disease, and diabetes.^3–6^ These include strict inclusion and exclusion criteria,^7,8^ insurance, lack of comfort, low clinical trial awareness, health literacy,^9^ religious beliefs, structural barriers,^6^ financial constraints,^10^ research misconduct, racism,^11^ and provider attitude.^12^

Substance abuse adds additional barriers to recruitment (concerns about social, legal, or employment consequences) and retention (stigma, relapse, ongoing legal involvement).^13^ The National Institute on Drug Abuse (NIDA) emphasizes the need for research focusing on improving treatment quality and outcomes among racial/ethnic minorities at high risk for substance use disorders (SUD). Black individuals with SUDs experience a significantly and disproportionately higher burden of SUD-related detrimental effects, including disabilities and deaths.^14–16^ Even though the National Drug Abuse Treatment Clinical Trials Network (CTN) has recruited over 5800 Black individuals into various CTN studies, some studies did not have sufficient sample size for Black participants, and those with sufficient sample size did not focus on treatment outcomes among Black individuals and investigate moderator or subgroup analysis focusing on race/ethnicity.^17^ CTN data also reveal differences in treatment entry,^17^ measurement equivalence,^18–20^ and treatment retention^17,21,22^ between Black individuals and other groups. Despite attempts to increase Black participant recruitment in large SUD trials, currently available data on the role of race in substance use treatment outcomes are still limited.^17^ These findings emphasize the need for developing methods to reduce barriers to recruitment and retention among Black individuals.

The Innovative Development of Research Engagement Manual (I-DREM) project was launched to direct efforts beyond documenting barriers toward developing a protocol to aid researchers from the planning stages through trial implementation to improve minority recruitment and retention. Although all racial/ethnic minority groups are generally underrepresented compared with White individuals in clinical trials, we focused on Black individuals due to the high burden of disease, delayed treatment entry, and reduced treatment retention among Black individuals. The goal of I-DREM was to develop sustainable solutions that would enhance the recruitment of Black individuals into SUD clinical trials and move toward making diversity in clinical trials a standard part of the SUD clinical research model. Here, we describe the I-DREM study design (three phases), the methodology of Phase 1 (focus groups and expert advisory board), themes identified in the literature review, and sample characteristics from Phase 1 focus groups.

## Methods

The I-DREM project (CTN-0109-A-1) is an extension effort of the CTN-funded study “Randomized, Placebo-Controlled Trial of Injectable Naltrexone and Monthly Injectable Buprenorphine for Cocaine Use Disorder (CURB-2; CTN-0109),” which seeks to use a combination of medications for the treatment of cocaine use disorder. The I-DREM study involved three phases, consisting of six steps to identify barriers, brainstorm all potential solutions, develop a manual, control quality, and revise the manual based on feedback and data. Phase 1 (development phase) focuses on conducting a literature search to identify barriers to participation, establishing an Expert Diversity Advisory Board (EDAB), and holding focus groups with patients. Phase 2 (evaluation phase) is designated for manual development and pilot feasibility testing in the CURB-2 study. Phase 3 (modification phase) focuses on obtaining feedback and satisfaction data and modifying the manual accordingly. Table 1 details the strategies and anticipated outcomes for each phase.

**Table 1.** Summary of Study

Phases	Phase 1: Development		Phase 2: Evaluation		Phase 3: Modification	
Steps	1.1 Identify barriers	1.2 Brainstorm solutions	2.1 Protocol development	2.2 Feasibility pilot	3.1 Delphi method	3.2 Modified protocol
Strategy	Literature searches/Focus groups and EDAB brainstorming	Focus groups and EDAB brainstorming	Feasible solutions are integrated into I-DREM for pilot among CURB-2 sites to enhance recruitment and retention of Black individuals	Disseminate solutions to the ongoing trial	Delphi method to solicit opinions from site research teams	Delphi voting results from stakeholders and EDAB to modify the protocol
Outcome	Identify and classify barriers	Identify solutions; address barriers; evaluate the feasibility of solutions		Quantitative # screened/enrolled Qualitative feedback on I-DREM	I-DREM utility and satisfaction evaluation	I-DREM available to pilot/use in prospective SUD trials
CURB-2 Study Timeline	Pre-implementation	Site selection/Training	Sites recruited ∼50% of participants	Sites continue recruitment	Recruitment wrap-up, study completion	Data analysis

CURB-2, Randomized, Placebo-Controlled Trial of Injectable Naltrexone and Monthly Injectable Buprenorphine for Cocaine Use Disorder; EDAB, Expert Diversity Advisory Board, I-DREM, Innovative Development of Research Engagement Manual; SUD, substance use disorders.

### Phase 1

We established the EDAB, which met to brainstorm potential barriers and solutions to the recruitment and retention of Black individuals. We reviewed the literature to identify barriers to clinical trial participation. Next, a series of questions and surveys were developed to obtain qualitative and quantitative feedback from stakeholder focus groups of Black individuals with SUD. The second step of Phase 1 was a brainstorming phase with the EDAB to develop a manual to enhance the recruitment and retention of Black participants in clinical trials. The University of Texas (UT) Southwestern Institutional Review Board (IRB) reviewed and approved the Phase 1 study (IRB # STU-2021-0917).

#### Expert Diversity Advisory Board

The EDAB was made up of 15 professionals and consisted of clinicians and researchers at collaborating sites who could provide guidance to facilitate the development of various iterations of the I-DREM manual, owners of nonprofit organizations that provided substance abuse services to individuals with current or a history of cocaine addiction, prominent leaders in the Black community, and people with relevant lived experience. These individuals had varying levels of education, training, and experience and united with I-DREM’s study team on strategic ways to develop, sustain, and retain research participants. The EDAB reviewed the literature and contributed to developing questions for focus groups and surveys.

The EDAB members were required to serve for at least 3 years and attend quarterly meetings remotely for 1–2 hours. Responsibilities included assisting in developing strategies for branding and messaging to disseminate information on SUD research and treatments to the public, providing feedback on written materials, facilitating strategies to enhance recruitment of ethnic minorities into clinical trials for SUD, facilitating connections between lead investigators and relevant community advocates and resources to fulfill the board’s purpose, training more professionals from ethnically diverse backgrounds, partnering with communities to complete specific projects, and nominating other potential board members. The EDAB discussed solutions to barriers and issues identified through the literature review and focus groups.

#### Literature review

We conducted a nonsystematic literature review with EDAB guidance to identify common themes that may contribute to the underrepresentation of Black individuals in substance abuse research. We identified mistrust, lack of diversity of providers and/or research teams, inconvenience due to time, location, and transportation, challenges with incentives, and poor communication as common themes. The study investigators and the EDAB considered these themes when constructing the focus group questions. A summary of each theme is presented below.

#### Mistrust

Mistrust of research investigators and institutions is the most significant attitudinal barrier to research participation reported by Black individuals.^23^ Black individuals were more likely than White individuals not to trust that their physician would explain a potential research study to them adequately and might not protect them from unnecessary risk in treatment.^24^ This etiology likely stems from historic events such as the Tuskegee Syphilis Study and the use of tissue from Henrietta Lacks without informed consent^25^ but may also be exacerbated by other social determinants of health and documented general health care disparities.^26,27^ Mistrust may stem from a lack of education about the realities and process of participating in research (randomized controlled trials or otherwise), negative encounters with health care providers, a personal history of unfair treatment and discrimination, provider attitudes, structural racism, research misconduct, concerns regarding the safety and efficacy of procedures, negative perceptions toward participating in research, delayed monetary-based incentives after completing studies, and other previous injustices.^3,11,26,28–35^ A feeling that the goals of research studies do not align with those of participants may also lead to feelings of mistrust.^36^

#### Lack of diversity of providers and/or research team

Incongruence in how researchers and participants view substance use, mental illness, treatment, and research may be a barrier to clinical trial participation. Cultural differences between researchers and participants may contribute to incongruent perspectives.^36^ A diverse study team and congruence in demographic features between researchers and participants are important for recruiting diverse samples.^37^ Diverse teams may be able to leverage greater trust, credibility, and understanding from the communities they intend to serve.^27,31,38–43^ However, the concept of researcher/participant congruence is ill-defined, and further work is needed to clarify how variable levels of congruence influence success in recruiting and retaining this population.

#### Inconvenience due to time, location, and transportation

Socioeconomic barriers that limit access to health care (unstable employment, access to health insurance, cost of treatments) may also limit enrollment into clinical trials.^24^ Disparities in transportation access may play a role in access to healthcare and research.^6,44–47^ These factors are likely to be relevant among substance-abusing populations, and there is evidence of significant health care access inequity by race.^48^ In addition to the cost of transportation, any interruption in daily activities could be problematic because of demanding and inflexible work schedules.^49^ Lack of available time off work may limit the ability to participate in many research studies where visits are conducted during normal working hours. Travel expenses, childcare, transportation, and travel time to research sites are important factors to consider when clinical research study teams craft recruitment plans.^6,50^

#### Challenges with incentives

The use of payment for clinical research participation is common and is one reason why many participants join studies. However, controversies remain about what constitutes an appropriate payment, with concerns about underpayment/nonpayment and overpayment (conferring an undue influence on an individual’s willingness to participate).^51,52^ Enrolling in a research study comes with associated costs and burdens (e.g., transportation, childcare arrangements, and time off from work) that may be more difficult to manage for those of lower socioeconomic status.^51^ This may effectively withhold necessary and/or novel treatments from those of low socioeconomic status. The Council for International Organizations of Medical Sciences emphasizes that participants should not have to pay for contributing to the social good of research.^53^ Thus, adequate pay and reimbursement of study-related costs may be important factors in overcoming barriers to study participation.

#### Poor communication

Misinformation due to researcher dishonesty, omission of important information, lengthy consent documents, insufficient outreach, or failure to explain the facts adequately has resulted in Black individuals being enrolled in studies without a genuine understanding of their participation.^23,31,34,54^ Research staff may manage the informed consent process in a way that covers the required material, but may not ensure full participant understanding.^55^ Poor health literacy and communication of scientific research findings to the public may lead to confusion about research and the research process,^23^ which may reduce participants’ willingness.

### Participants

#### Focus groups

Participants in the focus groups were consumer stakeholders with lived substance use experiences, especially cocaine use, who could provide feedback on factors affecting willingness to participate in SUD trials. They were required to self-report Black race, be age 18–90 years old, have a history of a SUD, be willing to participate in two virtual sessions, have access to a device for virtual meetings, provide informed consent, complete the study assessments in English, and report a desire to stop or reduce their illicit substance abuse. No exclusion criteria by gender were applied. Incarcerated persons were excluded from the study. The planned sample size was approximately 60 participants. All participants provided written informed consent before completing any study procedures. Participants were recruited via flyers distributed in the Dallas/Fort Worth region, direct referrals from current research participants, referrals from the EDAB, and referrals from regional non-profit organizations.

### Study procedures

#### Focus group methods

Potential participants were screened to ensure they met the eligibility criteria prior to signing the consent form for the study. Once eligibility was confirmed, before participating in the focus groups, participants were requested to answer several surveys about demographic characteristics, research knowledge, and alcohol and substance use history before participating in the focus groups. Participants were then assigned to a focus group and asked to attend two focus group sessions, which were conducted virtually to reduce barriers to participation and allow broader recruitment without geographic limitations. The second focus group was scheduled 1–3 weeks after the first focus group and depended on the availability and follow-up processes between the research staff and the participants.

Participant engagement in focus groups was via a secure virtual platform (Zoom) that was compliant with the Health Insurance Portability and Accountability Act and contained multiple authentication tools for meeting security and protection. Each focus group contained up to four members.

Focus groups were led by UT Southwestern facilitators using a topic-guided focus group interview guide that contained semi-structured questions based on previously identified barriers. Discussions focused on the willingness to join clinical trials of pharmacological interventions for substance use and factors that might encourage or discourage the participation of Black individuals. Table 2 identifies key barrier categories and sample questions or declarative statements that the facilitators could use to start and/or guide discussions in the focus groups. Most focus groups were 60–90 min, and participants had a choice during focus group sessions to remain on or off-camera, which enhanced confidentiality. Participants were assigned a unique study name/pseudonym during the focus groups to ensure anonymity. Focus group sessions were subsequently transcribed for future qualitative analyses.

**Table 2.** Barriers and Focus Group Questions

Theme	Sample questions
Lack of awareness	**The impact of clinical research on treatment, lack of perceived meaning or personal responsibility, unclear roles and responsibilities** Who should participate in clinical research? Why should people participate in clinical research? How does clinical research contribute to the dissemination of treatment? I would participate in a research study that offers me solutions for substance/drug abuse problem. Participating in research is everyone’s responsibility. Please describe why it is important for clinical trials to consist of participants from diverse ethnic backgrounds. Please describe your reaction about Black individuals participating in research studies. Research studies are meant to benefit me and my community. What do you think is the main reason why people should participate in research studies or projects?
Mistrust	**Understanding the value of clinical research, fear of the unknown, stigma of participation, communication styles from research team** What are the most critical pieces of clinical research? What would you expect from researchers? Before you decide to participate, what information would you like to know about the research? I have sufficient knowledge about participating in research studies. I trust that researchers will protect my health and my privacy. Would you be willing to include or invite a family member, friend, spouse, partner, girlfriend, or boyfriend in a similar research project? What would your initial reaction be if your family member, spouse, friend, boyfriend, girlfriend, or partner told you they were participating in a research study?
Lack of comfort with process	**Mistrust of process, fear of family’s or community’s opinion, lack of information, unresolved concerns regarding participation** What are the important steps of participation in research for researchers? What would you expect if you make a call to the research team in response to an ad? What are my obligations, responsibilities, and rights as a participant? I’m willing to share my information about my participation in any research study for my substance/drug abuse addiction.
Lack of information	**Lack of knowledge, information, or prior experience** What would you like to see from researchers? What do you believe is your role if you were a research participant? What do you believe is the researcher’s role in a trial? What do you think of the time involvement relative to the personal and scientific benefits? I trust that researchers will protect my health and my privacy What information is most important for you to know about a research study?
Time/Resources constraints	**Financial burden, time commitment, limitation barriers, logistics, compensation** How do you see the study fitting in your day-to-day life? What does it mean to you personally to be involved in this study? What could the study staff do to help you be more committed to completing the study? What reservations do you have about the study? To what degree does the compensation affect your decision? What about this study would be most difficult for you? Please describe some ways that would help you to remain in a research study from start to finish.

I-DREM staff and facilitators were trained on Zoom’s virtual platform in collecting and documenting specified data, including verbal informed consent, determining eligibility, and facilitating focus groups. All study personnel received Human Subjects Protection and Good Clinical Practice training. I-DREM focus group participants received $60 for the first completed session and $90 for the second.

### Measures

#### Demographics Form

The Demographics Form collected characteristics of the participants at screening, including sex, gender, date of birth, ethnicity, race, education, employment pattern, marital status, and information about the participant’s surrounding environment.

#### Research Knowledge Questionnaire

The Research Knowledge Questionnaire was developed by the study team to evaluate the level of knowledge the participant had about research. It was used before and after focus groups to evaluate changes in attitudes and knowledge acquisition related to willingness to participate in future research studies. Questions about research knowledge were on a 5-point Likert scale (strongly disagree, disagree, neither agree nor disagree, agree, and strongly agree). Additional questions were asked about why people might participate in research, opinions about research, and methods to improve recruitment and retention of Black participants.

#### Alcohol and Substance Use History

The Alcohol and Substance Use History form was completed at screening and queried whether the participant had ever used alcohol and/or various substances, and their age when the substance was first used. The intent of this measure was to document alcohol use, marijuana use, and illicit drug use (including prescription misuse). The questionnaire is part of The Substance Abuse and Addiction Collection of the Phen-X Toolkit Core Tier 1 (http://www.phenxtoolkit.org/),^56^ which includes substance use measures about age of onset, past 30-day quantity and frequency, and lifetime use for alcohol, tobacco, and other substances.

### Data management and statistical analysis plan

Study data was collected via REDCap. After data collection, the Data Team performed final data cleaning activities and locked the database from further modification. Descriptive statistical measures were completed for enrolled participants with and without completion status (i.e., those completing one rather than two focus groups).

## Results

Seventeen focus groups were conducted. Of the 65 adults screened for participation in the focus groups, 61 met the eligibility criteria and participated in at least one focus group. All participants were Black (non-Hispanic), and 45.9% (*n* = 28) were female. Most (*n* = 50, 82.0%) had completed high school or General Educational Development (GED) equivalent. Less than half of the sample was employed (*n* = 24, 29.3%), and almost one-third were disabled (*n* = 19, 31.1%). The mean age of the sample was 54.4 years. Over half of the sample reported that they or their family had access to health insurance (*n* = 39, 63.9%), and most (*n* = 57, 93.4%) reported having access to a means of transportation for work or medical visits. Sixteen (26.2%) participants reported participating in at least one prior study related to substance use prevention. Forty-eight participants attended a follow-up focus group and completed surveys.

Table 3 provides the frequencies and percentages of participant responses to survey questions. Unless otherwise noted, participants were limited to selecting one response to each question. Most focus group participants agreed that Black individuals are underrepresented in research studies (Table 1). Most participants did not express concerns about lack of knowledge and safety. However, the focus group participants considered a lack of knowledge about the research project, mistrust, safety concerns, and lack of transportation as reasons for not engaging with researchers (Table 1). The focus groups considered compensation, research benefits, study duration, privacy, safety, and side effects, equally important information to know before participation. The participants identified financial incentives, potential treatment options, and potential for improved awareness about substance abuse treatment as factors that determine retention in a study.

**Table 3.** Survey Questions and Response Frequencies

Survey question	Response frequency	
Do you feel that African Americans or persons of African heritage are well represented in research studies?	Yes	10 (16.4%)
	No	39 (63.9%)
	Unsure	3 (4.9%)
	I don’t know	9 (14.8%)
I have sufficient knowledge about participating in research studies.	Strongly agree	4 (6.6%)
	Agree	39 (63.9%)
	Neither agree or disagree	3 (4.9%)
	Disagree	15 (24.6%)
	Strongly disagree	0 (0.0%)
I trust that researchers will protect my health and my privacy.	Strongly agree	7 (11.5%)
	Agree	42 (68.9%)
	Neither agree or disagree	10 (16.4%)
	Disagree	2 (3.3%)
	Strongly disagree	0 (0.0%)
What do you think is the main reason why people should participate in research studies or projects?	For knowledge	20 (32.8%)
	For solutions	30 (49.2%)
	For treatment	10 (16.4%)
	I don’t know	1 (1.6%)
Please describe some reasons why you may decide not to speak with a researcher. (Select all that apply.)	Mistrust	23 (37.7%)
	Lack of knowledge about the research project	28 (45.9%)
	Lack of transportation	12 (19.7%)
	Safety concerns	17 (27.9%)
	I don’t know	21 (34.4%)
What information is the most important for you to know about a research study? (Select all that apply.)	Benefits of research	51 (83.6%)
	Compensation	49 (80.3%)
	Duration of study	41 (67.2%)
	Privacy concerns	46 (75.4%)
	Safety	53 (86.9%)
	Side effects of a trial medication or intervention	45 (73.8%)
Please describe some ways that would help you remain in a research study from start to finish. (Select all that apply.)	Financial incentives	58 (95.1%)
	Offer treatment options for participants	56 (91.8%)
	Offer more education about substance abuse treatment	48 (78.7%)
	I don’t know	1 (1.6%)
Do you use social media?	Yes	39 (63.9%)
	No	22 (36.1%)
How often do you log into your social media account?	Daily	19 (31.1%)
	Weekly	11 (18.0%)
	Bi-weekly	4 (6.6%)
	Monthly	5 (8.2%)
Do you use social media platforms for learning about different types of research studies?	Yes	32 (52.5%)
	No	29 (47.5%)
I would call a phone number in response to an advertisement on TV, Radio, Facebook, Instagram, Craigslist, Twitter, or Snapchat to enroll in a research study.	Strongly agree	0 (0.0%)
	Agree	41 (67.2%)
	Neither agree or disagree	11 (18.0%)
	Disagree	8 (13.1%)
	Strongly disagree	1 (1.6%)

## Discussion

I-DREM is a 3-phase study to identify barriers and develop a manual to increase the recruitment and retention of Black participants for clinical trials on SUD. Here, we report on the methodology and sample characteristics from Phase 1 of the project, which included a review of the literature, focus groups, and an expert advisory group to garner information needed for developing the manual. Sixty-one participants enrolled in the focus groups and provided quantitative data through survey responses and qualitative data from the focus group discussions. Survey responses indicated a positive view of research and a willingness to participate in research studies. Most focus group participants did not report passive concerns about lack of information and safety. It is possible that these themes may have been expressed more extensively in the focus group discussions, the qualitative analysis of which is underway. However, it is worth noting that when presented with reasons they may not want to talk to a researcher, roughly 40% of participants indicated that mistrust or a lack of research knowledge may be considerations, while approximately 30% indicated concerns about safety in research. These findings are not surprising, but they emphasize that particular importance should be placed on establishing an effective and trusting relationship between research staff and participants. Concerns about understanding the research purpose and procedures and participant safety also affect how informed consent procedures are conducted with participants. To overcome these barriers, study teams, providers, and the health care system must develop trustworthy reputations and focus on patient-centered care.

This study has limitations. First, the surveys were completed after study consent, so it is possible that the responses to the survey questions about research knowledge, communication about research information, and trust in the researchers were impacted by the fact that participants learned more about research through the consent process. Second, 26% of the participants had previously participated in research studies. Since the study aimed to identify barriers to research participation, having more participants with no prior research experience may have changed the results. Participants required access to a device to attend virtual meetings, possibly limiting the ability of some individuals to participate. Finally, the Research Knowledge Questionnaire created for this study has not been validated.

The next steps for the I-DREM project will be to analyze the qualitative data and then utilize the survey responses and qualitative data to develop a manual that researchers can use to increase recruitment and retention of Black participants with SUD in clinical trials. Phase 2 of the project will involve the development of the manual and initial testing of the manual. Phase 3 will involve the revision of the manual following feedback from research teams and the EDAB, completion of manual testing, and data analyses to evaluate the effectiveness of the manual for improving recruitment and retention of Black participants in a substance use clinical trial conducted as part of the CTN.

## Conclusion

I-DREM will outline concrete steps to aid researchers in the research planning stages. This resource can enhance cultural competency and facilitate the recruitment and retention of Black individuals into SUD clinical trials. Continued improvement in the recruitment of representative samples will be vital for decreasing disparities in clinical trials, improving the generalizability of findings, and enhancing the social benefits of research and health care.

## Abbreviations Used

CTN: Clinical Trials Network
CURB: Cocaine Use Reduction with Buprenorphine
EDAB: Expert Diversity Advisory Board
FDA: Food and Drug Administration
GED: General Educational Development
I-DREM: Innovative Development of Research Engagement Manual
IRB: Institutional Review Board
NCATS: National Center for Advancing Translational Sciences
NIDA: National Institute of Drug Abuse
NIMH: National Institute of Mental Health
SUD: Substance Use Disorder
